# Vegetated Ditches for the Mitigation of Pesticides Runoff in the Po Valley

**DOI:** 10.1371/journal.pone.0153287

**Published:** 2016-04-12

**Authors:** Stefan Otto, Salvatore E. Pappalardo, Alessandra Cardinali, Roberta Masin, Giuseppe Zanin, Maurizio Borin

**Affiliations:** 1 National Research Council, Institute of Agro-environmental and Forest Biology, Viale dell’Università 16, Legnaro (PD), Italy; 2 Department of Agronomy, Food, Natural Resources, Animals and Environment, University of Padova, Viale dell’Università 16, Legnaro (PD), Italy; Ghent University, BELGIUM

## Abstract

In intensive agricultural systems runoff is one of the major potential diffuse pollution pathways for pesticides and poses a risk to surface water. Ditches are common in the Po Valley and can potentially provide runoff mitigation for the protection of watercourses. The effectiveness depends on ditch characteristics, so there is an urgent need for site-specific field trials. The use of a fugacity model (multimedia model) can allows recognition of the mitigation main processes. A field experiment was conducted in order to evaluate the mitigation capacity of a typical vegetated ditch, and results were compared with predictions by a fugacity model. To evaluate herbicide mitigation after an extreme runoff, the ditch was flooded with water containing mesotrione, S-metolachlor and terbuthylazine. Two other subsequent floods with uncontaminated water were applied 27 and 82 days later to evaluate herbicides release. Results show that the ditch can immediately reduce runoff concentration of herbicides by at least 50% even in extreme flooding conditions. The half-distances were about 250 m. As a general rule, a runoff of 1 mm from 5 ha is mitigated by 99% in 100 m of vegetated ditch. Herbicides retention in the vegetated ditch was reversible, and the second flood mobilized 0.03-0.2% of the previous one, with a concentration below the drinking water limit of 0.1 μg L^-1^. No herbicide was detected in the third flood, because the residual amount in the ditch was too low. Fugacity model results show that specific physical-chemical parameters may be used and a specific soil-sediment-plant compartment included for modelling herbicides behaviour in a vegetated ditch, and confirm that accumulation is low or negligible for herbicides with a half-life of 40 days or less. Shallow vegetated ditches can thus be included in a general agri-environment scheme for the mitigation of pesticides runoff together with wetlands and linear buffer strips. These structures are present in the landscape, and their environmental role can be exploited by proper management.

## Introduction

Risk mitigation measures for pesticides are increasingly important [1]. Previous research [2] showed that a constructed surface flow wetland can reduce the pollution of watercourses from a watershed of hundreds of hectares in Northern Italy agro-systems. Indeed, in very fragmented landscapes such as in Po Valley there is also an urgent need for the mitigation of agricultural runoff from a large number of small farms in order to intercept pollutants before they enter a large watercourse, where mitigation is impossible.

Vegetated agricultural drainage ditches, hereafter ditches, are common in the Po Valley landscape, being a traditional part of field margins [3], and even if they are mainly designed for drainage purposes, they can provide two important ecosystem services: 1) habitats and green corridors for wildlife and wild plants [4], and 2) runoff mitigation for the protection of watercourses [5].

According to accepted classification, the ditch is an “off-field mitigation measure” for runoff as it can reduce flow velocity, intercept and remove sediment, organic material, nutrients and chemicals carried in runoff water. This has been shown in general [6], and for some studies the basic mitigation effectiveness is about 50% [7]. Yet mitigation depends strictly on ditch characteristics, i.e. size, length, slope, vegetation cover [8,9], macrophyte adsorption [10,11], and a great variability exists in ditch types and effectiveness.

As reported by [12] 98% of herbicide loss by runoff in the Po Valley is caused by a few extreme events with an estimated return period of 25–27 years, while 3–4 runoffs of low intensity are expected each spring-summer. However, in emerging climate change scenarios in which frequency of extreme rainfall events is estimated to increase locally [13], heavy runoff from croplands could represent a massive and uncontrollable non-point source threat to surface water bodies. Therefore, for the Po Valley there is an urgent need to do specific field measurements and gain insights into the main mitigation processes.

The fugacity model is a multi-media model that has proved to be very accurate in predicting concentrations of organic pesticides, both at field and watershed scale [14,15]. Its application could be very helpful to recognize and quantify the main pathway of environmental fate of pesticides in a little studied environment, so its application to a ditch is of interest.

The aim of the study was to assess in real field conditions the mitigation effect of a ditch for a simulated but realistic heavy runoff containing three of the main herbicides applied to maize in the Po Valley, and to highlight herbicides release after two subsequent floods with uncontaminated water. A simple fugacity model was applied to study the repartition of herbicides in the ditch, and predictions were compared to observations.

## Materials and Methods

### Site information and experiment layout

The trial was conducted on the Exp. Farm of Padova University (North-eastern Italy).

**Fig 1:**
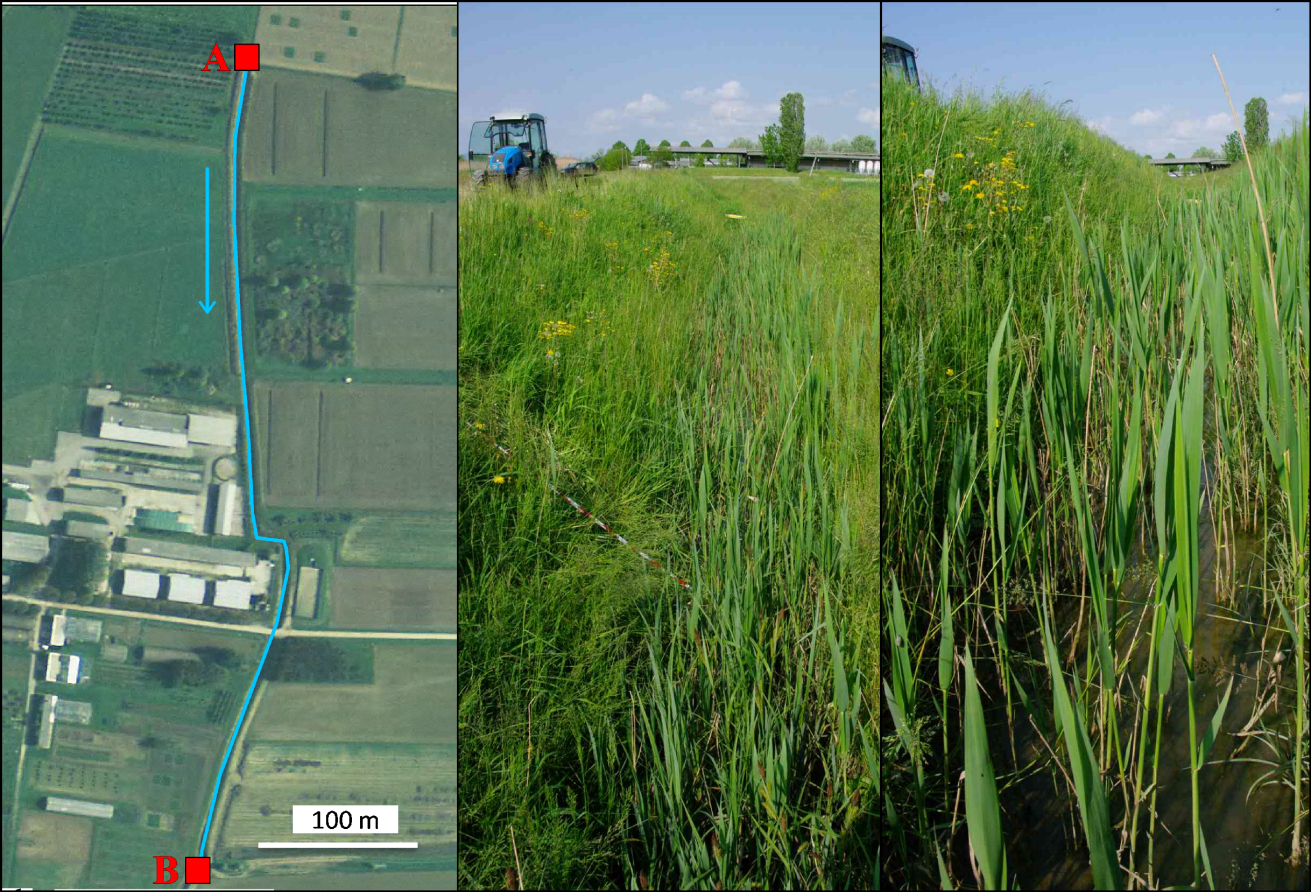
The experimental site. Left: the drainage network, with indication of inlet (A) and outlet (B) of the ditch and the direction of the flow; this image is similar but not identical to the original, and is therefore for illustrative purposes only. The Centre: overview of the ditch next to inlet. Right: detail of the flooded bed after the simulated runoff event (Photos: S. Otto and S.E. Pappalardo).

The studied ditch was 500 m long, of trapezoidal section (1 m bed, 2 m top, 1.8 m height), with a low slope (0.3%) designed as an irrigation and main drainage channel from a network of smaller ditches on 20 hectares of cropland where maize herbicides were not used in the previous cropping season (Fig 1).

The banks of the ditch were completely covered by vegetation 0.8–1.2 m tall, mainly perennial *Graminaceae*. Main species were *Dactylis glomerata*, *Convolvulus arvensis*, *Lolium multiflorum*, *Poa trivialis*, *Silene alba*, *Rumex crispus*, *Sonchus asper*, *Urtica dioica*, *Rubus* sp., *Bromus* sp., *Galium mollugo*, *Equisetum* sp., *Festuca arundinacea*, *Cynodon dactylon*. The bed part of the ditch was partially covered (10% of surface) by *Phragmites australis*, *Iris* sp., *Scirpus* sp., *Tipha* sp.

The estimated Manning's roughness coefficient of the ditch was 0.075, which is the median roughness coefficient for channels with dredged ditches covered by un-maintained weeds [16].

In ordinary conditions the ditch is without free water, and only after a rainfall of at least 20 mm a depth of 2–7 cm of water flows slowly to the outlet (0.2 m min^-1^).

In order to test the hydraulic performance of the ditch in an extreme runoff, a previous flood with uncontaminated water was conducted. About 50–55 m^3^ were necessary for the flooding, velocity of the water during flooding ranged from 0.07 (inlet) to 0.01 m sec^-1^ (outlet); after about 3 hours the flux at inlet was very low, less than 0.003 m sec^-1^ and about 35–40 m^3^ of water slowly passed the outlet in in the subsequent 10 hours. The ditch was therefore an open system that returns to its standard, dry, conditions in about 1 day.

On 24^th^ April 2015 a heavy runoff simulation was performed, and the ditch was flooded in 20 min with 52 m^3^ of water (corresponding to a flow of 156 m^3^/hour, or 2.6 mm/hour from a 6 ha basin) containing 60 g of the herbicide Lumax^®^, a common product for weed control in maize containing 37.5, 212.5, 187.5 g L^-1^ of mesotrione (CAS 104206-82-8), S-metolachlor (CAS 87392-12-9) and terbuthylazine (CAS 5915-41-3), respectively. Herbicide was regularly added to flood water in order to prevent a concentration peak moving through the ditch, and given that about 8 m^3^ of water were already in the ditch, the final concentration of the simulated runoff was 37, 213 and 188 μg L^-1^ of mesotrione, S-metolachlor and terbuthylazine, respectively.

These concentrations were about 100-fold higher than an ordinary runoff [12] to simulate an extreme runoff for both velocity and concentration of flow. The concentrations are similar to those observed for fungicides after exceptional rainfall in a study conducted in south-west Germany (7.0–83.4 μg L^-1^) [17].

The flood increased the water level by about 10 cm (mean value for the entire ditch). After 3 hours, 50 samples of free water and 10 samples of the saturated layer on the bed (mean depth: 5 cm) were collected at regular intervals from inlet to outlet. The bed sample included the suspended solids that precipitate within 3 hours, and hereafter called “sediment”. After 27 and 82 days the flooding was repeated with uncontaminated water, and sampling repeated in order to detect herbicides release.

For water concentration, the distance (m) required to reduce initial herbicide concentration by 50% was estimated (half-distance or D50).

### Calculation of runoff mitigation

The mitigation of runoff is calculated with the simple method suggested by [18]. For two values A and B of a quantitative parameter, with A greater than B, the percentage mitigation from A to B is:

M%=100*(A-B)/A (1)

For example, if at the ditch inlet (A, reference scenario) the mean concentration of a chemical is 12 μg L^-1^, and at the outlet (B, mitigated scenario) it is 3 μg L^-1^, the percentage mitigation from A to B is:

M%=100*(12-3)/12=75% (2)

### Analytical procedure

The procedures used for pesticide extraction and analysis derived from previous studies [19,20]. Details are in (S1 Text).

### The fugacity model

The fugacity model is a multimedia model that calculates the concentration of organic pesticides applied to a suitably modelled multi-compartment environment [21,22]. For chemicals used in agriculture a specific compartment for vegetation biomass was included by [23]. Repartition between compartments is based on partition coefficients of chemicals, fugacity capacity and volume of the compartments. The compartments are hypothesised as completely available, repartition is instantaneous, and the whole system in equilibrium. In field trials lasting hours, as in this study, this is the hardest condition to achieve. Nevertheless, differences between predicted and observed concentrations can highlight how far the system is from equilibrium and the main pathways and compartments involved in the repartition.

### Ditch modelling and repartition calculation

**Fig 2:**
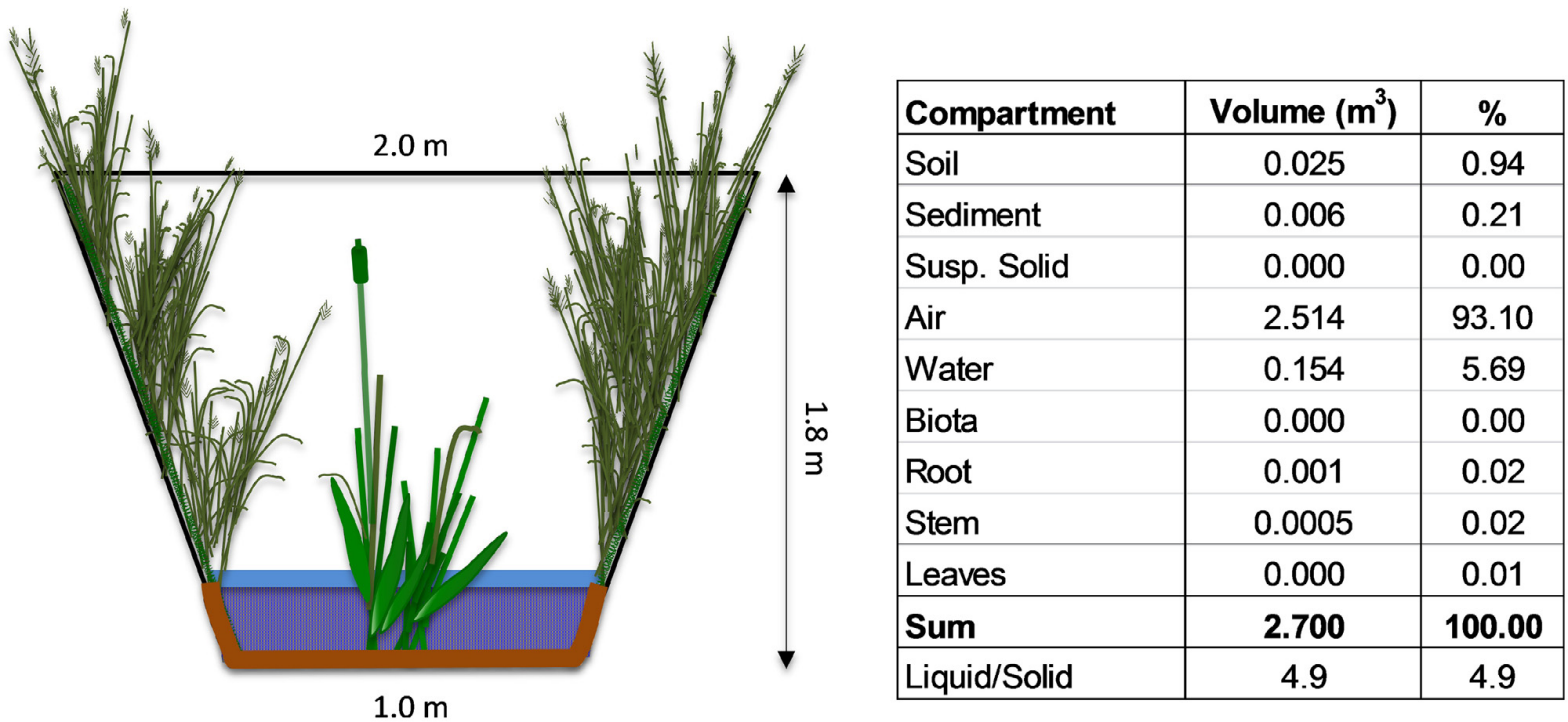
Scheme of the ditch after the flood, and modelling of 1-m length.

The flooded ditch was modelled in 9 environmental compartments (Fig 2), and repartition of the herbicides calculated for the three floods.

Even if initial soil moisture content was slightly different between floods, for simplicity the model of the ditch was the same for all floods (S1 Table). Therefore relative amount between compartments remained unchanged, and only concentration varied according to chemical load at flooding time. For the first contaminated flood the chemical load was the real amount applied, for the two subsequent uncontaminated floods the chemical load was calculated considering that after the flood the total amount of herbicides in the ditch decreased because: 1) part flowed out from outlet, 2) the rest degraded according to first-order kinetics.

## Results

### Water concentration after first flood (contaminated runoff)

**Fig 3:**
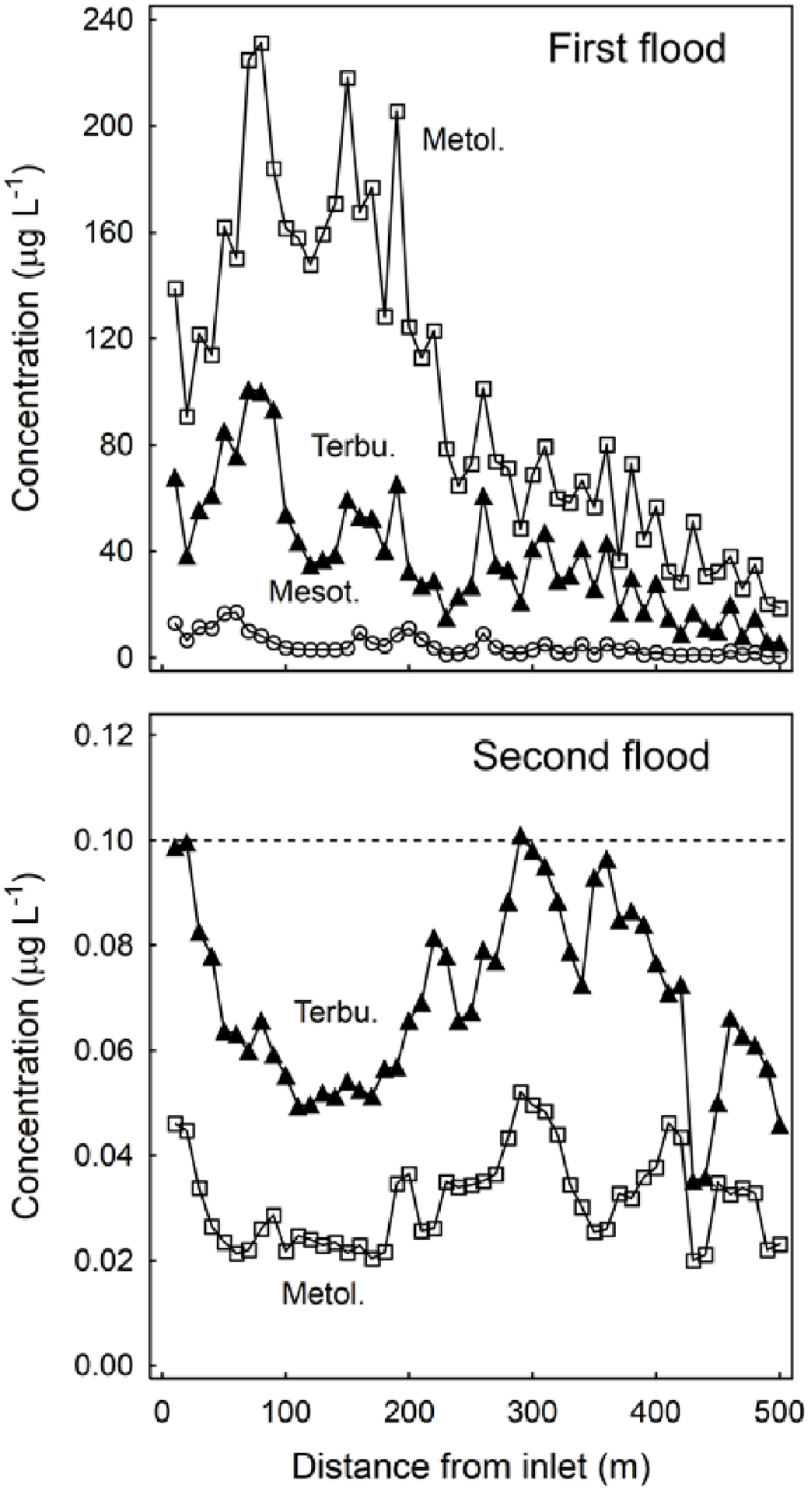
Concentration of herbicides in water in the first and second flood; all values in the third flood were below the detection limits. Mesotrione: empty circles; metolachlor: empty squares; terbuthylazine: full triangles. The lower axis is the distance from the inlet (m). The drinking water limit (0.1 μg L^-1^) is indicated. Detection limits: mesotrione = 0.070 μg L^-1^, S-metolachlor = 0.020 μg L^-1^, terbuthylazine = 0.014 μg L^-1^.

After complete flooding, concentration of the three herbicides was decreasing almost throughout the ditch, from inlet to outlet 500 m apart (Fig 3, top).

For the whole ditch, mean observed concentrations were 5, 99, 39 μg L^-1^ for mesotrione, S-metolachlor and terbuthylazine, respectively. The mean observed concentrations in water were lower than those applied, being about 12% for mesotrione, 47% for S-metolachlor and 19% for terbuthylazine. Sampling error was likely high because vegetation cover and water flow varied both along and across the ditch. Indeed, except for mesotrione, the observed values were very similar to that for atrazine (37%) obtained in a similar study by [24].

The highest concentrations of applied herbicides were found in the first 200 m of the ditch. The resistance to flow due to vegetation and the relevant length of the ditch, which is indeed a not rectilinear farm ditch with an open outlet (see Fig 1), hinder the achievement of a complete hydraulic equilibrium. At the outlet concentrations were lowest and the mitigation was 99%, 91% and 97% for mesotrione, S-metolachlor and terbuthylazine, respectively. These values are close to those obtained by [25] in similar conditions.

For the three herbicides the concentration was about half of the maximum (i.e. D50) at about 250 m from the inlet.

Taking into account concentrations weighted by the application rate, S-metolachlor concentration was on average 2.3-fold that of terbuthylazine, in accordance with S-metolachlor lower lipophilia. The S-metolachlor concentration was also 4-fold that of mesotrione, likely because 1) mesotrione is much more soluble and was rapidly transferred to the outlet, 2) mesotrione was promptly transformed into some metabolites, according to its 2–5 days dissipation half-life in basic soils, as in this study. It is worth noting that using unweighted concentrations, the ratio S-metolachlor/mesotrione would have been about 21, i.e. very misleading.

### Water concentration after second flood (first release)

The second flood 27 days after contamination caused a release of S-metolachlor and terbuthylazine from the ditch, while mesotrione was not detected (Fig 3, bottom). Concentrations of S-metolachlor and terbuthylazine were very low, close to the detection limit and almost uniform throughout the entire length of the ditch. Concentrations were below the drinking water limit (0.1 μg L^-1^), in this study used as fixed and prudent reference value; an adequate ecotoxicological endpoint for agricultural ditches may also be the predicted no effect concentration (PNEC). For the three herbicides under study the sensitive target in aquatic environment are Algae [26], and PNEC ranges from 0.1 to 350 μg L^-1^ so the risk is low. Average concentrations of S-metolachlor and terbuthylazine were 0.03 and 0.07 μg L^-1^ respectively, 3,500-fold lower for S-metolachlor and 500-fold lower for terbuthylazine with respect to first flood, i.e. mitigation of both herbicides was about 99.9% from first to second flood. Applying these same reduction ratios to mesotrione would result in a concentration of less than 0.01 μg L^-1^, well below the limit of detection (LOD) and in keeping with the lack of detections.

Concentration of S-metolachlor was regularly half or less that of terbuthylazine, and this is consistent with the physical-chemical characteristics: due to lower lipophilia, more S-metolachlor passed through the ditch in the first flood, and the rest almost dissipated before the second flood. This is in agreement with the relatively high persistence of terbuthylazine found in previous lab studies [27] and a field trial showing that about 30 days after application environmental load of terbuthylazine surpasses that of S-metolachlor [28].

### Water concentration after third flood (second release)

Herbicides were not detected in the third flood performed 55 days after the second. This result is consistent with that of the previous flood, when concentrations were already close to the detection limit.

### Sediment concentration

The content of the solid part in the saturated layer on the bed, i.e. the sediment in the ditch model, was on average 19% of the layer volume.

**Fig 4:**
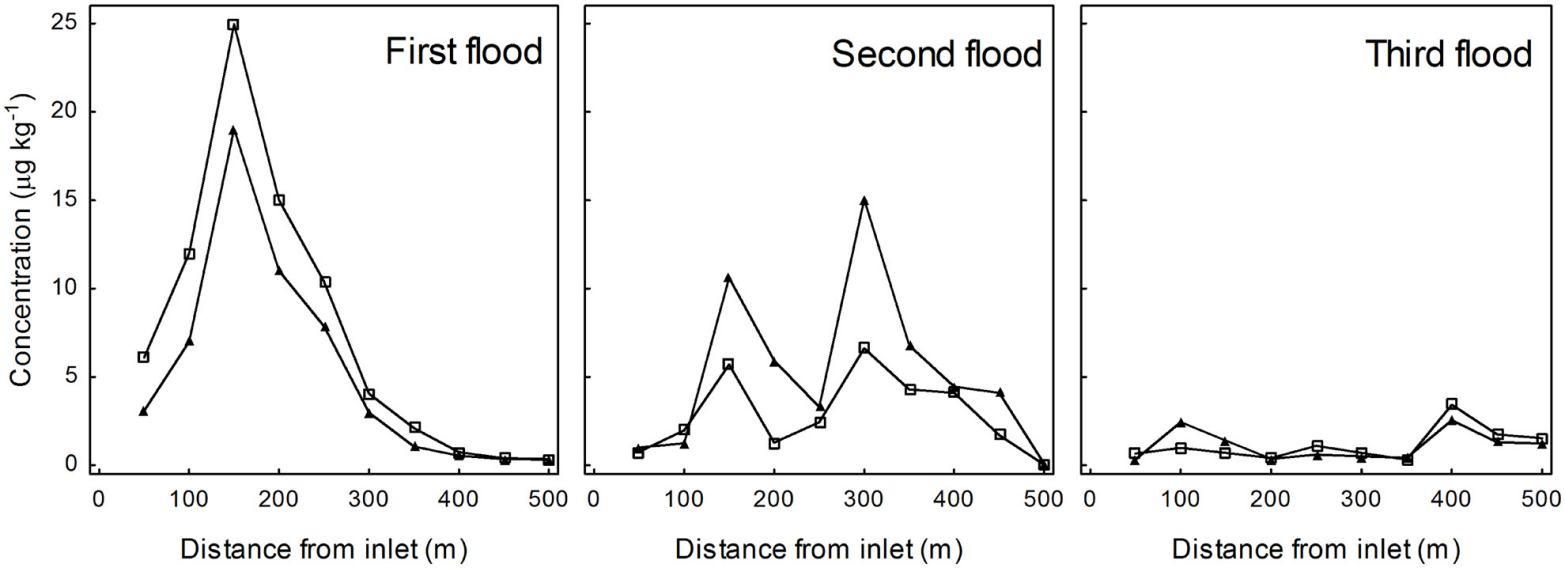
Concentration of herbicides in the dry sediment in the three floods. Metolachlor: empty squares; terbuthylazine: full triangles. The lower axis is the distance from the inlet (m). For mesotrione all values were below the detection limit. Detection limits: mesotrione = 0.070 μg kg^-1^, S-metolachlor = 0.020 μg kg^-1^, terbuthylazine = 0.014 μg kg^-1^

In the first flood, mean concentration in sediment was 10 and 7 μg kg^-1^ for S-metolachlor and terbuthylazine respectively, i.e. 5-10-fold lower than in water, and length-dependent (Fig 4).

Herbicides were detected in both water and sediment in all samples. Since they were applied to the ditch with water, this suggests that adsorption to the solid part of sediment begins promptly, and is likely complete when the ditch returns to standard dry conditions.

In the second flood, mean concentration in sediment was similar to the previous, 3 and 6 μg kg^-1^ for S-metolachlor and terbuthylazine respectively, but was 10-fold higher than in water. Concentration peak was shifted about 150 m nearer the outlet with respect to the first flood, according to the fact that in the first flood herbicides were added with water, in the second they were released from the bed by water.

This highlights, as for water, that environmental load of terbuthylazine surpasses S-metolachlor after 1 month, and confirms that the contamination source is the bed of the ditch.

In the third flood, the mean concentration in sediment was similar for both herbicides, about 1 μg kg^-1^, while herbicides were not found in water. This shows that after 82 days the total environmental load is low for both S-metolachlor and terbuthylazine.

Mesotrione was not found in any flood, in accordance with the low concentrations observed in water, again highlighting the reduced environmental load of this herbicide after application.

### Physical-chemical parameters selection

The main physical-chemical parameters of the applied herbicides were selected from the literature. Molecular weight, solubility, vapour pressure and K_OW_ were taken from [26], whereas, because several values of K_OC_ were reported, a selection was made (S2 Table).

For mesotrione a wide range of K_OC_ sorption coefficients is available because adsorption is directly correlated with soil organic carbon [26,29,30]; since organic matter in the ditch is estimated at about 2% (S1 Table), the highest K_OC_ reported by [26] (390 L kg^-1^) was selected. For S-metolachlor the value of 118 L kg^-1^ reported by [31] was selected since it was obtained with vegetation organic matter and is very similar to the value of 123 L kg^-1^ obtained by [32] in a topsoil with 2.6% of organic carbon. For terbuthylazine the average value in the literature was selected (259 L kg^-1^).

### Fugacity model results

93% of the ditch volume is air, but the studied herbicides have low vapour pressure, so concentration in air is negligible (S3 Table). Even if the banks of the ditch were completely covered by vegetation, only the strip submerged by the flood is considered in the simulation, so the total vegetation biomass is only about 0.05% of total volume; repartition in this compartment occurs for 0.1% (mesotrione) to 4.0% (S-metolachlor and terbuthylazine) of applied amount, and accumulation is negligible. As a consequence, the solid (soil, sediments and suspended solids) and liquid compartments (free flowing water, water in the fluid layer on ditch bed, water in soil) were of most importance for repartition. Even if the liquid is about 4-fold the solid, the model calculates that 44–73% of applied herbicides is adsorbed onto the solid part and 26–51% dissolved in water. This highlights the magnitude of adsorption for these chemicals.

For the first flood, the concentrations calculated in water by the fugacity model were very close to those observed. For the first flood, predicted vs. observed values were 7 vs. 5, 86 vs. 99, 49 vs. 39 μg L^-1^ for mesotrione, S-metolachlor and terbuthylazine respectively; the model was therefore very precise for water.

Considering that 37 m^3^ of the contaminated flood passed the outlet, 10–22% of applied herbicides left the ditch with this. The remaining part degrades, and first order kinetics calculates that 4%, 32% and 51% of applied mesotrione, S-metolachlor and terbuthylazine respectively were in the ditch at the time of the second (uncontaminated) flood. This highlights the importance of degradation for these herbicides.

For the second flood, the concentration calculated in water by the fugacity model was 0.25 μg L^-1^ for mesotrione, while observed concentrations were below the LOD of 0.070 μg L^-1^. For S-metolachlor and terbuthylazine the fugacity model calculated concentrations of 24 and 21 μg L^-1^, respectively, in accordance with the residual amount before flood. These values were 300-900-fold higher than those observed, so the model was very imprecise for water.

First order kinetics calculates that 65–99% of herbicides still in the ditch after the second flood are degraded before third. In particular, the residual amount of mesotrione is nearly zero (about 0.1 mg). The chemical load in the third flood was therefore low.

For the third flood, the concentrations calculated in water by the fugacity model were 0.0004, 3.0 and 6.3 μg L^-1^ for mesotrione, S-metolachlor and terbuthylazine, respectively. Observed values were all below the LOD. Considering the LOD as “observed values” for comparison purposes, calculated values were 150-500-fold higher than observed, so the model was again very imprecise for water.

The fugacity model calculates in the first and second flood a concentration of S-metolachlor and terbuthylazine in sediment 2-70-fold higher than observed. This suggests that: 1) for the first flood full repartition is not achieved in the 3-hours sampling period; 2) for the second mobilisation (desorption) with flowing water is significant but incomplete, likely requiring days to finish.

It is possible that this steady overestimation in both water and sediments is caused by the absence of a specific “soil-sediment-plant” compartment in the model.

After the third flood, the fugacity model calculates an average concentration in the solid compartment of the ditch (soil and sediments) of about 0.005 μg kg^-1^ for mesotrione, and 12–54 μg kg^-1^ for S-metolachlor and terbuthylazine, corresponding to about 1.5 g of total herbicides in the whole ditch (500 m^2^), a mass 1000-fold lower than a standard herbicide application on 1 ha of crop. This shows that herbicide accumulation is negligible, so debris from ditch maintenance is not toxic for crops.

## Discussion

Even after one regular and intense input of herbicides, concentrations in water in the ditch were lower than input and quite regularly decreasing along the ditch, showing that repartition begins soon and is very effective, because the mean concentration detected in water was only 10–45% of that applied, and at the outlet the concentration was mitigated by 90–99% according to the calculation method suggested by [18]. This highlights the importance of adsorption onto the sediment-soil-plant complex, and the ditch length or the residence time (hydraulic retention).

According to results from specific runoff studies, 3–4 ordinary runoff events are expected every year in the Po Valley, each with a load of about 0.2 g ha^-1^ of metolachlor and terbuthylazine [12].

In this study, the ditch was flooded with about 12 g of those herbicides, corresponding to the herbicide loss from 60–70 ha of treated cropland, and a 500 m long and 1 m bed wide ditch provided mitigation of 90–99%. In brief, for ordinary runoff, mitigation of at least 90% can be achieved with 10 m^2^ of ditch/hectare of cropland. This highlights that ditches can be very effective for the mitigation of ordinary runoff, and that mitigation is similar to that obtained with vegetative filter strips (86–88% for S-metolachlor and terbuthylazine [33]).

It is of interest to compare mitigation effectiveness of a ditch and wetland for heavy or extreme runoff events.

According to [2], the mitigation effectiveness of a constructed surface flow wetland for a heavy runoff of 3.5 mm from a 10 ha basin is 90% for each 50 m in length for a 15 m wide wetland, corresponding to the weighted value of 75 m^2^ of wetland/hectare of cropland.

In the present study, taking into account concentrations at the outlet, 500 m of ditch 1 m bed wide provided mitigation of about 95% for a runoff of 52 m^3^, corresponding to 3.5 mm runoff from 1.5 ha. For 90% mitigation (500/1.5)*90/95 = 318 m^2^ of ditch/hectare of cropland are thus necessary. This weighted value is about 4-fold that of wetland, suggesting that a ditch is much less efficient, but it is reasonable because a runoff of 3.5 mm is extreme and the residence time in the ditch much shorter than in the wetland, where the laminar sheet flow enhances pesticide interception [17]. It is worth noting that for a realistic runoff of 1 mm from 5.2 ha, 99% mitigation can be achieved with 100 m of ditch/hectare of cropland. This result can be summarized with the mitigation rule “1 mm from 5 ha is mitigated by 99% (M) in 100 m of vegetated ditch 1 m bed wide”. For example, for a runoff containing 1 μg L^-1^ of herbicide (A), application of this rule results in a concentration (B) at the outlet of:

$$\text{B}=-((\text{M}*\text{A}/100)-\text{A})=-((99*1/100)-1)=0{.01\;\mu \text{g L}}^{\text{-1}}$$ (3)

For pesticides, mitigation effectiveness depends on many physical and chemical processes, degradation, sedimentation, infiltration and adsorption onto plant surfaces, the relative importance of which is not completely known. The role of adsorption coefficient and solubility of pesticides has still to be clarified, as also reported by [5], and in wetlands there are even cases of great mobility of pesticide with high K_OC_ [34]. Most information is still based on model simulations, i.e. with SWAT [16], so there is a need for field trials.

Sampling error is likely high in field conditions, and a recent study show that changes in macrophyte biomass and particulate/dissolved organic carbon levels caused concentration variations of several orders of magnitude in space, especially for highly hydrophobic chemicals [35]. Nevertheless, the results of the present study show that when a contaminated runoff is convoyed to a ditch covered by semi-natural vegetation, and the linear flow is about 3 m min^-1^, a length of 250 m is enough for halving the initial concentration just by means of adsorption of the pesticide onto the sediment-soil-plant complex. For lower, and more realistic, flow velocity the half-length dissipation would be similarly lower.

When the ditch has an open outlet, in the case of heavy runoff events there is an immediate risk of transferring a runoff contaminated above the drinking water limit (0.1 μg L^-1^) to surface waters. In the Po Valley this risk can be managed in two ways: 1) by insertion of a sediment pond after the outlet; 2) closing the outlet of the ditch, which practically becomes a linear constructed surface flow wetland. Implementation of this second option is anyway not easy and not often suggested because during heavy rainfall the primary role of ditches is to quickly remove water from fields to prevent flooding.

Adsorption onto the sediment-soil-plant complex is quickly reversible, and successive floods can mobilize herbicides according to their dissipation dynamics in soil and sediments. Observed concentrations were anyway very low, below the drinking water limit, showing that the ditch is an effective structure for trapping herbicides.

The lack of detection in the third flood 82 days after contamination highlights the low persistence of these chemicals in ditches, where dissipation half-lives can differ from those in agricultural soil. For example, [36] reported for S-metolachlor a half-life of 12 days for microbiologically active soils, while the mean generic value reported by [26] is 28 days. The vegetation and the periphyton [34] could play a relevant role in stimulating biological degradation, and the inclusion of experimental dissipation values for ditches would improve precision of dissipation dynamic modelling, but further research is needed for this.

The results of the fugacity model depend on some inevitable assumptions. Comparison of the model’s outcome with observations for subsequent simulations can test the goodness of choices because the model should always succeed or fail, and provide information about relative magnitude of pathways. For a ditch the model highlights the importance of K_OC_ sorption coefficient and half-life of pesticides in flood concentration and mid-term release. When the calculation aims to estimate predicted environmental concentrations, there is a need for specific values of pesticides adsorption onto the sediment-soil-plant complex since 1) sorption depends on type and age of organic matter [31]; 2) the K_OC_ likely varies in time, as recently observed in studies conducted on a river [37]. When K_OC_ is about 200–400 L kg^-1^ the pesticide is mainly adsorbed and release from ditches is low. When the half-life is 3-5 weeks, accumulation is very unlikely. A runoff with 100–200 μg L^-1^ convoyed to a ditch can be practically decontaminated in 3–4 months. Furthermore, the model results suggest that when contaminated runoff enters a ditch, two subsequent stages occur: 1) solubilisation stage, when risk to surface water depends mainly on chemicals solubility and flow velocity; 2) repartition stage, when environmental load is driven by sorption and dissipation from the sediment-soil-plant complex. The inclusion of this compartment in the model would improve general precision of repartition calculation.

Debris from regular dredging and vegetation management of the ditch are calculated as non-toxic for crops, and can be spread on cropland before ploughing. The entire cycle of depuration with ditches is then of low impact and cost. Developing countries in particular could gain advantage from this low-cost and easily-implemented system [38].

Given that combinations of mitigation measures can be very effective [18], a sustainable scheme for the mitigation of pesticide runoff to surface water can be based on: 1) ditches for the immediate mitigation of direct runoff from fields; 2) wetlands, serving a watershed of hundreds of hectares; 3) linear buffer strips along water courses of high quality.

Vegetated ditches are already present on cropland, and their environmental and ecosystem services can be exploited by proper management and maintenance.

## Conclusions

Vegetated ditches have a great herbicides runoff mitigation potential for the protection of watercourses and can be inserted in environmental schemes. Their effectiveness with shallow flooding is high and length-dependent. In typical ditches of North-eastern Italy, for the main pre-emergence herbicides applied in maize, the distance required to reduce initial concentration by 50% is about 250 m. As a general rule for herbicides with K_OC_ of 110–400 L kg^-1^, a runoff of 1 mm from 5 ha is mitigated by 99% in 100 m of vegetated ditch 1 m bed wide.

The dissipation of herbicides in ditches is site-specific and mainly due to degradation and adsorption, while outflow with water discharge is low since the flood is shallow. Coverage of emergent plants and the hydraulic residence time is of great importance, and a better insight into herbicides adsorption onto the sediment-soil-plant complex is needed.

## Supporting Information

S1 Text Analytical procedure. (DOCX)

S1 Table Modelling of the vegetated ditch. (DOCX)

S2 Table Physical-chemical parameters of applied herbicides. (DOCX)

S3 Table Calculation of herbicides repartition in the vegetated ditch using the fugacity model. (DOCX)

S1 Dataset Original data. (XLSX)

## References

[1] European Commission. Directive 2009/128/EC of the European Parliament and of the Council of 21 October 2009 establishing a framework for Community action to achieve the sustainable use of pesticides.

[2] Pappalardo SE, Otto S, Gasparini V, Zanin G, Borin M. Mitigation of herbicide runoff as an ecosystem service from a constructed surface flow wetland. Hydrobiologia, 2015; 10.1007/s10750-015-2375-1

[3] Hackett M, Lawrence A. Multifunctional role of field margins in arable farming. CEA report. Cambridge Environmental Assessments; 2104 –ADAS UK Ltd., Battlegate Road, Boxworth, Cambridge, CB23 4NN, UK.

[4] Herzon I, Helenius J. Agricultural drainage ditches, their biological importance and functioning. Biol. Cons. 2008;141: 1171–1183. 10.1016/j.biocon.2008.03.005

[5] Vymazal J, Březinová T. The use of constructed wetlands for removal of pesticides from agricultural runoff and drainage: A review. Environ. Int. 2015;75: 11–20. 10.1016/j.envint.2014.10.026 25461411

[6] Bennett ER, Moore MT, Cooper CM, Smith S, Shields FD, Drouillard KG, et al Vegetated agricultural drainage ditches for the mitigation of pyrethroid-associated runoff. Environ. Toxicol. Chem. 2005;24: 2121–2127. 16193737 10.1897/04-357r.1

[7] Gregoire C, Elsaesser D, Huguenot D, Lange J, Lebeau T, Merli A, et al Mitigation of agricultural nonpoint-source pesticide pollution in artificial wetland ecosystems. Environ. Chem. Lett. 2009;7: 205–231. 10.1007/s10311-008-0167-9

[8] Bouldin JL, Farris JL, Moore MT, Cooper C.M. Vegetative and structural characteristics of agricultural drainages in the Mississippi Delta landscapes. Environ. Poll. 2004;132: 403–411. 10.1016/j.envpol.2004.05.026 15325456

[9] Moore MT, Denton DL, Cooper CM, Wrysinski J, Miller JL, Reece K, et al Mitigation assessment of vegetated drainage ditches for collecting irrigation runoff in California. J. Environ. Qual. 2008:37: 486–493. 10.2134/jeq2007.0172 18268312

[10] Hand LH, Kuet SF, Lane MCG, Maund SJ, Warinton JS, Hill IR. Influences of aquatic plants on the fate of the pyrethroid insecticide lambda-cyhalothrin in aquatic environments. Environ. Toxicol. Chem. 2001;20: 1740–1745. 10.1002/etc.5620200817 11491557

[11] Merlin G, Vuillod M, Lissolo T, Clement B. Fate and bioaccumulation of isoproturon in outdoor aquatic microcosms. Environ. Toxicol. Chem. 2002;21: 1236–1242. 12069308

[12] Cardinali A, Otto S, Zanin G. Herbicides runoff in vegetative filter strips: evaluation and validation of a recent rainfall return period model. Int. J. Environ. Analyt. Chem. 2013;1: 1–10. 10.1080/03067319.2013.841151

[13] Zollo LA, Rillo V, Bucchignani E, Montesarchio M, Mercogliano P. Extreme temperature and precipitation events over Italy: assessment of high resolution simulations with COSMO-CLM and future scenarios. Int. J. Climatol. 2015;in press.

[14] Di Guardo A, Calamari D, Zanin G, Consalter A, Mackay D. A fugacity model of pesticide runoff to surface water: Development and validation Chemosphere 1994;28: 511–531 10.1016/0045-6535(94)90295-X

[15] Ghirardello D, Morselli M, Otto S, Zanin G, Di Guardo A. Investigating the need of complex vs. simple scenarios to improve predictions of aquatic ecosystem exposure with the SoilPlus model. Environ. Poll. 2014;184: 502–510. 10.1016/j.envpol.2013.10.002 24172657

[16] Zhang X, Zhang M. Modeling effectiveness of agricultural BMPs to reduce sediment load and organophosphate pesticides in surface runoff. Sci. Total Environ. 2001;409: 1949–1958. 10.1016/j.scitotenv.2011.02.012 21377192

[17] Bereswill R, Burkhard G, Streloke M, Schulz R. Entry and toxicity of organic pesticides and copper in vineyard streams: Erosion rills jeopardise the efficiency of riparian buffer strips. Agr. Ecosyst. Environ. 2012;146: 81–92. 10.1016/j.agee.2011.10.010

[18] Otto S, Loddo D, Baldoin C, Zanin G. Spray drift reduction techniques for vineyards in fragmented landscapes. J. Environ. Manage. 2015:162: 290–298. 10.1016/j.jenvman.2015.07.060 26265598

[19] Freitas LG, Götz CW, Ruff M, Singer HP, Müller SR. Quantification of the new triketone herbicides, sulcotrione and mesotrione, and other important herbicides and metabolites, at the ng/l level in surface waters using liquid chromatography-tandem mass spectrometry. J. Chromatogr. A 2004;1028: 277–286. 10.1016/j.chroma.2003.11.094 14989481

[20] Barchanska H, Rusek M, Szatkowska A. New procedures for simultaneous determination of mesotrione and atrazine in water and soil. Comparison of the degradation processes of mesotrione and atrazine. Environ. Monit. Assess. 2012;184: 321–334. 10.1007/s10661-011-1970-5 21416215

[21] Mackay D, Paterson S. Fugacity revisited. Environ. Sci. Technol. 1982;16: 654A–660A. 10.1021/es00106a724 22646890

[22] MacLeod M, Scheringer M, McKone TE, Hungerbuhler K. The state of multimedia mass-balance modeling in environmental science and decision-making. Environ. Sci. Technol. 2010;44: 8360–8364. 10.1021/es100968w 20964363

[23] Calamari D, Vighi M, Bacci E. The use of terrestrial plants biomass as a parameter in the fugacity model. Chemosphere 1987;16: 2359–2364. 10.1016/0045-6535(87)90293-1

[24] Moore MT, Bennet ER, Cooper CM, Smith S Jr, Shields FD Jr, Milam CD, et al Transport and fate of atrazine and lambda-cyhalothrin in an agricultural drainage ditch in the Mississippi Delta, USA. Agr. Ecosyst. Environ. 2001;87: 309–314.

[25] Cooper CM, Moore MT, Bennet ER, Smith S, Farris JL, Milam CD, et al Innovative use of vegetated drainage ditches for reducing agricultural runoff. Water Sci. Technol. 2004;49: 117–123. 15053106

[26] MacBean C. The Pesticide Manual, 16th Edition, 2012 British Crop Protection Council Publications Alton, Hampshire, UK.

[27] Fava L, Orrù MA, Scardala S, Funari E. Leaching potential of carbamates and their metabolites and comparison with triazines. Microchem. J. 2007;86: 204–208. 10.1016/j.microc.2007.03.003

[28] Otto S, Vianello M, Infantino A, Zanin G, Di Guardo A. Effect of a full-grown vegetative filter strip on herbicide runoff. Maintaining of filter capacity over time. Chemosphere 2008;71: 74–82. 10.1016/j.chemosphere.2007.10.029 18045643

[29] Chaabane H, Vulliet E, Calvayrac C, Coste C-M, Cooper J-F. Behaviour of sulcotrione and mesotrione in two soils. Pest Manage. Sci. 2008;64, 86–93. 10.1002/ps.1456 17912682

[30] Tomlin CDS. The Pesticide Manual, 14th Edition, 2006 British Crop Protection Council Publications, Alton, Hampshire, UK.

[31] Aslam S, Garnier P, Rumpel C, Parent SE, Benoit P. Adsorption and desorption behavior of selected pesticides as influenced by decomposition of maize mulch. Chemosphere 2013;91: 1447–1455. 10.1016/j.chemosphere.2012.12.005 23434076

[32] Laabs V, Amelung W, Pinto A, Altstaedt A, Zech W. Leaching and degradation of corn and soybean pesticides in an Oxisol of the Brazilian Cerrados. Chemosphere 2000;41: 1441–1449. 11057581 10.1016/s0045-6535(99)00546-9

[33] Otto S, Cardinali A, Marotta E, Paradisi C, Zanin G. Effect of vegetative filter strips on herbicide runoff under various types of rainfall. Chemosphere 2012;88: 113–119. 10.1016/j.chemosphere.2012.02.081 22463948

[34] Tournebize J, Passeport E, Chaumont C, Fesneau C, Guenne A, Vincent B. Pesticide de-contamination of surface waters as a wetland ecosystem service in agricultural landscapes. Ecol. Eng. 2013;56: 51–59. 10.1016/j.ecoleng.2012.06.001

[35] Morselli M, Semplice M, De Laender F, Van den Brink PJ, Di Guardo A. Importance of environmental and biomass dynamics in predicting chemical exposure in ecological risk assessment. Sci. Total Environ. 2015;526: 338–345. 10.1016/j.scitotenv.2015.04.072 25967479

[36] Barra Caracciolo A, Giuliano G, Grenni P, Guzzella L, Pozzoni F, Bottoni P, et al Degradation and leaching of the herbicides metolachlor and diuron: a case study in an area of Northern Italy. Environ. Poll. 2005;134: 525–534. 10.1016/j.envpol.2004.08.014 15620598

[37] Boithias L, Sauvage S, Merlin G, Jean S, Probst J-L, Sánchez Pérez JM. New insight into pesticide partition coefficient Kd for modelling pesticide fluvial transport: Application to an agricultural catchment in south-western France. Chemosphere 2014;99: 134–142. 10.1016/j.chemosphere.2013.10.050 24275149

[38] Mahabali S, Spanoghe P. Mitigation of two insecticides by wetland plants: feasibility study for the treatment of agricultural runoff in Suriname (South America). Water Air Soil Pollut. 2014;225:1771 10.1007/s11270-013-1771-2

