# Supplementary material for: Vegetated Ditches for the Mitigation of Pesticides Runoff in the Po Valley
Source: PLoS One. 2016 Apr 12;11(4):e0153287. doi: 10.1371/journal.pone.0153287 (PMC4829255; doi:10.1371/journal.pone.0153287)
Supplement: S1 Text — (DOCX) [file pone.0153287.s001.docx]

# Supporting Information

## S1 Text. Analytical procedure

The procedures used for pesticide extraction and analysis derived from previous studies. Herbicide standards: terbuthylazine (99.5% purity), metolachlor (98.0% purity) and mesotrione (99.5% purity) were purchased by Dr. Ehrenstorfer (Augsburg, Germany). Methanol (HPLC-grade), water (HPLC-grade) and all other chemicals (analytical-grade) were purchased from Sigma-Aldrich. All analytes with individual stock solutions were prepared in methanol with concentration of 0.1 mg mL^–1^. Mixtures of standard solutions were prepared in concentration ranges from 0.1 to 2.5 μg mL^–1^. They were used as spiked solutions for sample fortification and for calibration curves.

To determine herbicide concentration in water, 1 L field samples were filtered at room temperature with a vacuum equipment using cellulose nitrate membrane filters 0.45 μm pore size. After filtration an acetate buffer (2.5 M) was added (ca. 1%, v/v) and water samples were extracted using a TELOS *neo*^TM^ PRP polymeric SPE column (60 mg, Kinesis) and a J.T. Baker SPE-12G glass column processor at a flow rate of 15 mL min^–1^. After extraction the analytes were eluted with 5 mL of methanol without the vacuum and methanol aliquots were reduced to approximately 50 μL with the use of a nitrogen gas stream at 45 °C.

To determine herbicide concentration in sediment, 20 g of dry sediment were weighed in dark bottles and 50 mL of methanol-0.1 M HCl (9-1, v/v) was added. The bottles were shaken for an hour at 200 rpm at room temperature (about 18 °C). Mixtures were then centrifuged for 15 min at 3,200 rpm (Heraeus Christ Labofuge GL, Germany) and supernatant filtered at 0.45 μm pore size. The filtrate was then evaporated at 38 °C using an IKA^®^ RV 8 rotary evaporator ((IKA^®^, Werke GmbH & C., Staufen, Germany) and the residue was dissolved in 50 mL of 0.1 M HCl. Subsequently, the extraction was conducted. The conditions of solid-phase extraction were the same as for water samples.

To define the analyte recoveries, water and sediment (without analytes) were spiked with herbicides at concentrations of 0.4, 1.0 and 2.2 μg mL^–1^. Extraction was then the same as reported above. The recoveries in water were: mesotrione and metolachlor 95%, terbuthylazine 91%; the sediment recoveries were: mesotrione 81%, metolachlor 76%, terbuthylazine 79%.

The analyses were performed by LC-MS using a 1100 Series Agilent Technologies system (CA, US), equipped with binary pump, diode array detector, and MSD SL Trap mass spectrometer with ESI source. A Eurospher II (Knauer, Berlin, Germany) column C18 P with TMS endcapping, 150 × 4.6 mm i.d., 3 μm, 110 Å was used to analyze the samples, the mobile phase consisted of 0.6% formic acid in water (solvent A) and methanol (solvent B).

Gradient elution programme: from 0 to 4 min a linear increase of solvent B from 60% to 80% and flow rate from 0.4 to 0.6 mL min^–1^, from 4 to 11 min a linear increase of solvent B from 80% to 100% at flow rate of 0.6 mL min^–1^; initial conditions were re-established in 5 min and re-equilibration time was 2 min.

A 10 μL sample volume was manually injected each time. Retention times were 5.5 (±0.2), 10.1 (±0.3) and 9.0 (±0.2) min for mesotrione, metolachlor, and terbuthylazine respectively.

The limit of detection (LOD), 3:1 signal-to-noise ratio, was 0.070 μg L^‑1^ for mesotrione, 0.020 μg L^‑1^ for S-metolachlor, and 0.014 μg L^‑1^ for terbuthylazine.
