# Supplementary material for: Vegetated Ditches for the Mitigation of Pesticides Runoff in the Po Valley
Source: PLoS One. 2016 Apr 12;11(4):e0153287. doi: 10.1371/journal.pone.0153287 (PMC4829255; doi:10.1371/journal.pone.0153287)
Supplement: S1 Table — (DOCX) [file pone.0153287.s002.docx]

# Supporting Information

## S1 Table. Modelling of the vegetated ditch.

| **Parameter** | **Value** | **Note** |
| --- | --- | --- |
| Bed width (m) | 1 |  |
| Top width (m) | 2 |  |
| Height (m) | 1.8 |  |
| Length (m) | 500 |  |
| Volume of water in the flood (m^3^) | 52 | (1) |
| Part of flood increasing the saturated layer on the bed (%) | 20 | (2) |
| Height of the saturated layer on the bed (cm) | 3 | (3) |
| Water in the saturated layer on the bed (%) | 81 | (4) |
| Vegetation biomass on the bank (kg/m^2^) | 2 | (5) |
| Vegetation biomass density (kg/m^3^) | 0.8 | (6) |
| Ratio (Veg. biom. in the bed)/(Veg. biom. in the bank) | 0.6 | (7) |
| Height of the saturated soil layer (cm) | 4 | (8) |
| Water in the saturated soil layer (%) | 50 | (9) |
| Slope of the ditch (%) | 0,1 | (10) |
| Suspended solids in the free water (m^3^) | 0.052 | (11) |
| Volume of Biota in the whole ditch (m^3^) | 0 | (12) |
| Part of the free water passing the outlet (%) | 90 | (13) |
| Organic carbon content in the soil (accessible part) (%) | 2 | (14) |
| Organic carbon content in suspended solid and sediment (%) | 4 | (14) |

**Notes**

(1) Volume of water used in each of the three floods.

(2) Part of the flooding water increases the saturated layer of the bed, i.e. the fluid layer composed of sediment and water; the rest becomes free water flowing slowly to outlet and beyond.

(3) Height of the accessible part of the saturated layer, mean of the entire ditch length; height ranges from 1 cm (inlet) to 10 cm (outlet). The selected height of 3 cm is about half the observed, but is assumed to be the part completely accessible to chemicals during the 3 hours of sampling.

(4) Liquid part in the saturated layer, percentage of volume. This is an experimental value, average of 30 samples for the determination of herbicides in sediment.

(5) Green biomass of plants on the bank, kg m^‑2^. This is an experimental value, average of 10 sampling areas of 1 m^2^ selected along the entire ditch length. In the fugacity model accumulation differs between root, stem and foliage. In the present simulation it has been assumed that aerial biomass is given 40% by leaves and 60% by stems, and that roots are 67% of total aerial biomass.

(6) Biomass density according to [1].

(7) Plant cover on the bed and on the banks was uniform along the ditch, the cover of the bank was regularly higher than on the bed; the relative cover was assessed visually.

(8) Height of the soil layer saturated by flooding water, on both the bed and the banks, assumed to be completely accessible by chemicals. Each flood increases the water level by about 10 cm (mean value for the entire ditch), then the full length of the saturated soil covering the ditch is given by:

Length=100 cm of bed width+2*3 cm height of the fluid layer+2*10 cm level free water=126 cm.

(9) Water-filled pore space in soil was set at 50% according to results in a specific study [2].

(10) The slope of the ditch is low as is common in Po Valley, and free flowing water is only observed after heavy rainfall.

(11) The free flowing water had a variable content of suspended solids from inlet to outlet, being higher at about 200 m from inlet; the selected mean value was 1 L of suspended solids each 1,000 L of water.

(12) The original fugacity model includes the animal biomass (Biota), for example fish, because chemicals can accumulate in fat; in the present simulation this compartment is included with nil volume in order to retain the original structure and ease its inclusion when needed.

(13) According to field observation, about 90% of the flood passed the outlet and removed part of the chemicals; this advection reduced the chemical load in the vegetated ditch after each flood and consequently poses the highest potential risk for watercourses.

(14) On the Exp. Farm of Padova University the organic content in field soil is 0.92% [3], but is estimated as higher in the soil under the ditch, sediments and suspended solids according to specific studies [4].

**References for S2 Table.**

1. Calamari D, Vighi M, Bacci E. The use of terrestrial plants biomass as a parameter in the fugacity model. Chemosphere 1987;16: 2359-2364. doi: 10.1016/0045-6535(87)90293-1
2. Ullah S, Faulkner SP. Denitrification potential of different land-use types in an agricultural watershed, lower Mississippi valley. Ecol. Eng. 2006;28: 131-140. doi: 10.1016/j.ecoleng.2006.05.007
3. Otto S, Cardinali A, Marotta E, Paradisi C, Zanin G. Effect of vegetative filter strips on herbicide runoff under various types of rainfall. Chemosphere 2012; 88: 113-119. doi: 10.1016/j.chemosphere.2012.02.081
4. Balestrini R, Arese C, Delconte CA, Lotti A, Salerno F. Nitrogen removal in subsurface water by narrow buffer strips in the intensive farming landscape of the Po River watershed, Italy. Ecol. Eng. 2011;37: 148-157. doi: 10.1016/j.ecoleng.2010.08.003
