# Supplementary material for: Vegetated Ditches for the Mitigation of Pesticides Runoff in the Po Valley
Source: PLoS One. 2016 Apr 12;11(4):e0153287. doi: 10.1371/journal.pone.0153287 (PMC4829255; doi:10.1371/journal.pone.0153287)
Supplement: S2 Table — (DOCX) [file pone.0153287.s003.docx]

# Supporting Information

## S2 Table. Physical-chemical parameters of applied herbicides

| Parameter | Unit | Mesotrione | Reference | S-metolach. | Reference | Terbuthylaz. | Reference |
| --- | --- | --- | --- | --- | --- | --- | --- |
| CAS | # | 104206-82-8 | [1] | 87392-12-9 | [1] | 5915-41-3 | [1] |
| Applied rate | kg/ha | 0.15 | Rec. rate in maize | 1.25 | Rec. rate in maize | 0.75 | Rec. rate in maize |
| Mol. weight | g/M | 339.30 | [1] | 283.80 | [1] | 229.70 | [1] |
| Solub. in water | g/L | 15 | [1] | 0.480 | [1] | 0.009 | [1] |
| Vapor press. | Pa | 0.00569 | [1] | 0.004 | [1] | 0.00009 | [1] |
| LogK_OW_ | L/kg | 0.11 | [1] | 3.05 | [1] | 3.40 | [1] |
|  |  |  |  | 3.13 | [2] | 3.04 | [3] |
|  |  |  |  | 3.00 | [4] | 3.20 | [4] |
|  |  | **0.11** | **Selected value** | **3.05** | **Selected value** | **3.40** | **Selected value** |
| K_OC_ | L/kg | 19-141 | [4] | 121-309 | [4] | 162-378 | [4] |
|  |  | 80 | Mean [4] | 215 | Mean [4] | 270 | Mean [4] |
|  |  | 19-390 | [1] | 61-369 | [1] | 162-333 | [1] |
|  |  | 210 | Mean [1] | 215 | Mean [1] | 248 | Mean [1] |
|  |  |  |  | 83-118 | [5] |  |  |
|  |  |  |  | 101 | Mean [5] |  |  |
|  |  |  |  | 123 | [6] |  |  |
|  |  | 145 | General Mean | 163 | General Mean | 259 | General Mean |
|  |  | **390** | **Selected value** | **118** | **Selected value** | **259** | **Selected value** |
| t50 in field | days | 3-7 | [4] | 11-30 | [4] | 30-60 | [4] |
|  |  | 5 | Mean [4] | 21 | Mean [4] | 45 | Mean [4] |
|  |  | 2-14 | [1] | 6-49 | [1] | 6.5-149.8 | [1] |
|  |  |  | Mean [1] | 28 | Mean [1] | 78 | Mean [1] |
|  |  |  | Median [1] | 30 | Median [1] | 17 | Median [1] |
|  |  |  | [7] | 12 | [7] | 20 | [7] |
|  |  |  |  | 12 | [8] | 35 | [9] |
|  |  |  |  | 23 | [3] | 45 | [3] |
|  |  |  |  | 14 | [10] | 27 | [10] |
|  |  | 5 | General Mean | 21 | General Mean | 38 | General Mean |
|  |  | **5** | **Selected value** | **21** | **Selected value** | **38** | **Selected value** |

**References for S3 Table.**

1. MacBean C. The Pesticide Manual, 16th Edition, 2012. British Crop Protection Council Publications. Alton, Hampshire, UK.
2. Mackay D, Shiu WY, Ma KC. Illustrated handbook of physical-chemical properties and environmental fate for organic chemicals. Volume V: Pesticide Chemicals. 1997. Lewis Publisher, Boca Raton, USA.
3. Di Guardo A, Calamari D, Zanin G, Consalter A, Mackay D. A fugacity model of pesticide runoff to surface water: Development and validation. Chemosphere 1994;28: 511–531. doi: 10.1016/0045-6535(94)90295-X
4. Tomlin CDS. The Pesticide Manual, 14th Edition, 2006. British Crop Protection Council Publications, Alton, Hampshire, UK.
5. Aslam S, Garnier P, Rumpel C, Parent SE, Benoit P. Adsorption and desorption behavior of selected pesticides as influenced by decomposition of maize mulch. Chemosphere 2013;91: 1447–1455. doi: 10.1016/j.chemosphere.2012.12.005
6. Laabs V, Amelung W, Pinto A, Altstaedt A, Zech W. Leaching and degradation of corn and soybean pesticides in an Oxisol of the Brazilian Cerrados. Chemosphere 2000;41: 1441-1449.
7. Otto S, Cardinali A, Marotta E, Paradisi C, Zanin G. Effect of vegetative filter strips on herbicide runoff under various types of rainfall. Chemosphere 2012;88: 113-119. doi: 10.1016/j.chemosphere.2012.02.081
8. Barra Caracciolo A, Giuliano G, Grenni P, Guzzella L, Pozzoni F, Bottoni P, Fava L, Crobe A, Orrù M, Funari E. Degradation and leaching of the herbicides metolachlor and diuron: a case study in an area of Northern Italy. Environ. Poll. 2005;134: 525-534. doi: 10.1016/j.envpol.2004.08.014
9. Fava L, Orrù MA, Scardala S, Funari E. Leaching potential of carbamates and their metabolites and comparison with triazines. Microchem. J. 2007;86: 204-208. doi: 10.1016/j.microc.2007.03.003
10. Vianello M, Vischetti C, Scarponi L, Zanin G. Herbicide losses in runoff events from a field with a low slope: role of a vegetative filter strip. Chemosphere 2005;61: 717-725. doi: 10.1016/j.chemosphere.2005.03.043
