# Supplementary material for: Vegetated Ditches for the Mitigation of Pesticides Runoff in the Po Valley
Source: PLoS One. 2016 Apr 12;11(4):e0153287. doi: 10.1371/journal.pone.0153287 (PMC4829255; doi:10.1371/journal.pone.0153287)
Supplement: S3 Table — (DOCX) [file pone.0153287.s004.docx]

# Supporting Information

## S3 Table. Calculation of herbicides repartition in the vegetated ditch using the fugacity model

| Chemical | **Mesotrione** | **S-Metolach.** | **Terbuthyl.** |
| --- | --- | --- | --- |
| Molecular weight | 339.30 | 283.80 | 229.70 |
| Solubility in water (g/l) | 15.000 | 0.480 | 0.009 |
| Solubility in water (M/m^3^) | 44.209 | 1.691 | 0.039 |
| Vapor pressure (Pa) | 5.69E-03 | 3.70E-03 | 9.00E-05 |
| Lipophilia: logKow (L/kg) | 0.11 | 3.05 | 3.21 |
| T (°C) | 25 | 25 | 25 |
| R (gas constant) (Pa*m^3^)/(M*°K) | 8.3136 | 8.3136 | 8.3136 |
| Half-life (t50) (days) | 5 | 21 | 38 |
| **Density of compartments (Di) (kg/m^3^)** |  |  |  |
| Soil: Ds | 1500 | | |
| Sediment: Dsed | 1500 | | |
| Suspended solid: Dss | 1500 | | |
| Air: Da | 1.19 | | |
| Water: Dw | 1000 | | |
| Biota: Db | 1000 | | |
| Root: Dro | 800 | | |
| Stem: Dst | 800 | | |
| Foliage: Dfo | 800 | | |
| **Moles introduced (n)** | 0.007 | 0.045 | 0.049 |
| **Mass introduced (g)** | 2.25 | 12.75 | 11.25 |
| **Volumes of compartments (Vi) (m^3^)** |  |  |  |
| Soil: Vs | 12.680 | | |
| Sediment: Vsed | 2.850 | | |
| Susp. Solid: Vss | 0.052 | | |
| Air: Va | 1256.893 | | |
| Water: Vw | 76.830 | | |
| Biota: Vb | 0.000 | | |
| Roots: Vro | 0.279 | | |
| Stems: Vst | 0.250 | | |
| Leaves: Vle | 0.166 | | |
| Sum(Vi) | 1350.000 | | |
| **Partition coefficients** |  |  |  |
| **Soil** |  |  |  |
| Soil: Koc (L/kg) | 390.00 | 118.00 | 259.00 |
| Soil: Koc (m^3^/kg) | 3.900E-01 | 1.180E-01 | 2.590E-01 |
| %OCsoil=%OMsoil | 2.00 | | |
| Kp=Koc*%OC/100 (L/kg) (=Cs/Cw) | 7.805E+00 | 2.361E+00 | 5.183E+00 |
| **Soil (Cs/Cw): Kp (m^3^/kg)** | **7.805E-03** | **2.361E-03** | **5.183E-03** |
| **Sediment** |  |  |  |
| %OCsed | 4.00 | | |
| Ksed=Koc*%OCsed/100 (L/kg) (=Csed/Cw) | 15.60 | 4.72 | 10.36 |
| **Sediment (Csed/Cw): Ksed (m^3^/kg)** | **1.560E-02** | **8.600E-03** | **1.036E-02** |
| **Susp. Solid** |  |  |  |
| %OCss | 4.00 | | |
| Kss=Koc*%OCss/100 (L/kg) (=Css/Cw) | 15.60 | 4.72 | 10.36 |
| **Susp. solid (Css/Cw): Kss (m^3^/kg)** | **1.560E-02** | **8.600E-03** | **1.036E-02** |
| **Air** |  |  |  |
| **Air: Ka (M/pa*mc) (=1/RT)** | **4.0342E-04** | **4.0342E-04** | **4.0342E-04** |

| **Water** |  |  |  |
| --- | --- | --- | --- |
| **Water: Kw=H=VP/S (Pa*m^3^/M)** | **1.287E-04** | **2.188E-03** | **2.297E-03** |
| Air/Water (Ca/Cw): Kaw=H' adim.=(H/RT) | **5.192E-08** | **8.825E-07** | **9.267E-07** |
| **Biota (Aquatic biom.)** |  |  |  |
| LogBCF (0.85*logKow-0.7) | -0.61 | 1.89 | 2.03 |
| BCF (dmc/kg) (=L/kg) (=Cb/Cw) | 2.475E-01 | 7.807E+01 | 1.068E+02 |
| **Biota (Cd/Cw): BCF (m^3^/kg)** | **2.475E-04** | **7.807E-02** | **1.068E-01** |
| **Root** |  |  |  |
| RCF (dmc/kg)=10^(0.77*logKow-1.52)+0.82 | 8.567E-01 | 7.558E+00 | 9.767E+00 |
| **Root: RCF (m^3^/kg)** | **8.567E-04** | **7.558E-03** | **9.767E-03** |
| **Stem** |  |  |  |
| SCF (dmc/kg)=(10^(0.95*logKow-2.05)+0.82)*0.784*EXP(-((logKow-1.78)^2)/2.44) | 2.078E-01 | 3.181E+00 | 3.665E+00 |
| **Stem: SCF (m^3^/kg)** | **2.078E-04** | **3.181E-03** | **3.665E-03** |
| **Foliage** |  |  |  |
| Zw=1/H (M/pa*mc) | **7769.537** | **457.117** | **435.350** |
| Kaw=H' adim.=(H/RT) (=Ca/Cw) | 5.192E-08 | 8.825E-07 | 9.267E-07 |
| LogKaw | -7.28 | -6.05 | -6.03 |
| **Foliage: FCF (m^3^/kg)**=10^(-1.61+logKow-logKaw))/Da | **5.118E+05** | **2.623E+07** | **3.610E+07** |
| **Coefficients summary** |  |  |  |
| Solubility in water (g/l) | 1.500E+01 | 4.800E-01 | 9.000E-03 |
| Vapor pressure (Pa) | 5.690E-03 | 3.700E-03 | 9.000E-05 |
| Lipophilia: Kow (m^3^/kg) | 1.288E-03 | 1.122E+00 | 1.622E+00 |
| Soil: Koc (m^3^/kg) | 3.900E-01 | 1.180E-01 | 2.590E-01 |
| Soil (Cs/Cw): Kp (m3/kg) | 7.805E-03 | 2.361E-03 | 5.183E-03 |
| Sediment (Csed/Cw): Ksed (m^3^/kg) | 1.560E-02 | 4.720E-03 | 1.036E-02 |
| Susp. solid (Css/Cw): Kss (m^3^/kg) | 1.560E-02 | 4.720E-03 | 1.036E-02 |
| Air: Ka (M/pa*mc) (=1/RT) | 4.034E-04 | 4.034E-04 | 4.034E-04 |
| Air/Water (Ca/Cw): Kaw=H' adim.=(H/RT) | 5.192E-08 | 8.825E-07 | 9.267E-07 |
| Water: Kw=H=VP/S (Pa*m^3^/M) | 1.287E-04 | 2.188E-03 | 2.297E-03 |
| Biota (Cd/Cw): BCF (m^3^/kg) | 2.475E-04 | 7.807E-02 | 1.068E-01 |
| Root: RCF (m^3^/kg) | 8.567E-04 | 7.558E-03 | 9.767E-03 |
| Stem: SCF (m3/kg) | 2.078E-04 | 3.181E-03 | 3.665E-03 |
| Foliage: FCF (m^3^/kg) | 5.118E+05 | 2.623E+07 | 3.610E+07 |
| **Calculation of Zi [M/(m^3^*Pa)]** |  |  |  |
| Soil: Zs=Kp*Ds*Zw | 9.096E+04 | 1.619E+03 | 3.385E+03 |
| Sediment: Zsed=Ksed*Dsed*Zw | 1.818E+05 | 3.236E+03 | 6.765E+03 |
| Susp. solid: Zss=Kss*Dss*Zw | 1.818E+05 | 3.236E+03 | 6.765E+03 |
| Air: Za=Ka=1/RT | 4.034E-04 | 4.034E-04 | 4.034E-04 |
| Water: Zw=1/Kw=1/H | 7.770E+03 | 4.571E+02 | 4.354E+02 |
| Biota: Zb=BCF*Db*Zw | 1.923E+03 | 3.569E+04 | 4.649E+04 |
| Root: Zro=RCF*Dro*Zw | 5.325E+03 | 2.764E+03 | 3.402E+03 |
| Stem: Zst=SCF*Dst*Zw | 1.292E+03 | 1.163E+03 | 1.277E+03 |
| Foliage: Zfo=FCF*Za | 2.065E+02 | 1.058E+04 | 1.456E+04 |
| Sum(Zi) | 4.711E+05 | 5.874E+04 | 8.308E+04 |

| **Calculation of Zi*Vi (M/Pa)** |  |  |  |
| --- | --- | --- | --- |
| Soil | 1.153E+06 | 2.053E+04 | 4.292E+04 |
| Sediment | 5.182E+05 | 9.224E+03 | 1.928E+04 |
| Susp. Solid | 9.454E+03 | 1.683E+02 | 3.518E+02 |
| Air | 5.071E-01 | 5.071E-01 | 5.071E-01 |
| Water | 5.969E+05 | 3.512E+04 | 3.345E+04 |
| Biota | 0.000E+00 | 0.000E+00 | 0.000E+00 |
| Root | 1.484E+03 | 7.703E+02 | 9.482E+02 |
| Stem | 3.224E+02 | 2.904E+02 | 3.186E+02 |
| Foliage | 3.436E+01 | 1.760E+03 | 2.424E+03 |
| Sum(Zi*Vi) | 2.280E+06 | 6.787E+04 | 9.969E+04 |
| **Calculation of Fugacity (Pa): f=n/Sum(Zi*Vi)** | 2.909E-09 | 6.620E-07 | 4.913E-07 |
| **Concentration (μg/L)=f*Zi*PM*1000** |  |  |  |
| Soil | 8.98E+01 | 3.04E+02 | 3.82E+02 |
| Sediment | 1.79E+02 | 6.08E+02 | 7.63E+02 |
| Susp. Solid | 1.79E+02 | 6.08E+02 | 7.63E+02 |
| Air | 3.98E-07 | 7.58E-05 | 4.55E-05 |
| Water | 7.67E+00 | 8.59E+01 | 4.91E+01 |
| Biota | - | - | - |
| Root | 5.26E+00 | 5.19E+02 | 3.84E+02 |
| Stem | 1.27E+00 | 2.19E+02 | 1.44E+02 |
| Foliage | 2.04E-01 | 1.99E+03 | 1.64E+03 |
